# Supplementary material for: Urban resources limit pair coordination over offspring provisioning
Source: Sci Rep. 2020 Sep 28;10:15888. doi: 10.1038/s41598-020-72951-2 (PMC7522258; doi:10.1038/s41598-020-72951-2)
Supplement: Supplementary file 1 — Supplementary file1 [file 41598_2020_72951_MOESM1_ESM.docx]

**Urban resources limit pair coordination over offspring provisioning**

Davide Baldan^1*^, Jenny Q Ouyang^1^

^1^ Department of Biology, University of Nevada, Reno, Reno, NV USA

^*^ To whom correspondence should be addressed: Department of Biology, University of Nevada, Reno, 1664 N Virginia St., Reno, NV 89557. Telephone: 775-784-6089 Email: [dbaldan@unr.edu](mailto:dbaldan@unr.edu)

Keywords: house wren, synchrony, alternation, parental care, arthropod, rural

**Abstract**

The amount of care parents provide to the offspring is complicated by an evolutionary conflict of interest (‘sexual conflict’) between the two parents. Recent theoretical models suggest that pair coordination of the provisioning may reduce this conflict and increase parent and offspring fitness. Despite empirical studies showing that pair coordination is common in avian species, it remains unclear how environmental and ecological conditions might promote or limit the ability of parents to coordinate care. We compared the level of pair coordination, measured as alternation and synchrony of the nest visits, of house wrens *Troglodytes aedon* pairs breeding in a rural (10 nests) and a suburban (9 nests) site and investigated how differences in parental behaviours were related to habitat composition, prey abundance and how they ultimately related to reproductive success. We found that parents alternated and synchronized their nest visits more in the rural site compared to the suburban one. The suburban site is characterized by a more fragmented habitat with more coniferous trees and less caterpillar availability. Offspring from the rural site were heavier at fledging than at the suburban site. Taken together, these results suggest that environmental conditions play an important role on the emergence of coordinated parental care and that considering environmental variables is pivotal to assess the fitness consequences of parental strategies.

Keywords: coordination, alternation, synchrony, parental care, caterpillar, house wren

**Introduction**

Offspring of altricial species are heavily dependent on parental care at early life stages^1^. While parental care provides direct fitness benefits to both offspring and parents, care behaviour is costly for parents and often leads to reduced parental survival and future reproduction^2^. In species with biparental care, the total amount of care each parent contributes is also affected by a conflict of interest (‘sexual conflict’) between the two parents^3^ because each parent is selected to exploit its mate by providing a smaller share of the care^3,4^. A central goal in evolutionary biology is to understand how this conflict is resolved and whether parents can reach a cooperative agreement over how much to care for offspring^5,6^.

Theoretical models have been widely used over the past decades to investigate the evolutionary outcome of sexual conflict^7-10^. Despite the variety with which the dynamic of parental investment was modelled, including either fixed (“sealed bids”)^7^ or repeated (“negotiated”)^8,10,11^ bouts of investment, these models have consistently shown that sexual conflict and negotiation between the parents lower parental and offspring fitness^10^, *i.e.,* each carer withholds part of its potential investment to avoid being exploited by the partner^12^. Recently however, it has been proposed that forms of pair coordination over offspring care, more precisely alternation^13,14^ and synchrony of the provisioning^15,16^, promote parental cooperation and increase parent and offspring fitness^15-17^. Specifically, alternation and synchrony of the nest visits are patterns of nest provisioning resulting from active behavioural interactions between the parents^13-16^. Alternation of nest visits occurs when a visit by one parent at the nest is followed by a visit of its partner (e.g. MFMFMFMF) because parents take turns of visits over time^13,14^. This pattern of visits has been proposed to increase total parental investment because by taking turns, parents continuously stimulate each other to provision at their maximum rate^13^ (unlike a situation in which parents make runs of consecutive feedings, *e.g.* MMMFFF). Synchrony of the nest visits occurs instead when parents visits the nest together to feed the offspring because they forage in the same patch^16^ and actively wait for the partner to return together to the nest^15^. This synchrony of feeds has been shown to reduce nest predation^18^ (by decreasing the time parents spend at the nest site) and increase offspring survival (via a more equal partitioning of food to the offspring)^15,19^. These synchronized visits contrast with a pattern of regular visits by independent parents (*e.g.* each parent regularly visits the nest alone over time), because in the latter case the time that at least one parent is at the nest site increases (making the nest more conspicuous for predators) and there is increased likelihood that food items get monopolized by a few young^19^.

Pair coordination of the provisioning has been the topic of intense recent research in avian species (see Savage and Hinde^20^ for a review on the topic). Despite some studies having investigated biological factors (*e.g.* parental workload^16,21,22^, offspring need during development^23^) affecting pair coordination, a remaining gap in knowledge is how environmental and ecological conditions might limit the ability of parents to coordinate care^24,25^. In particular, a study on a great tit (*Parus major*) population in a deciduous forest has shown that pairs in close proximity and recorded on the same day have similar levels of coordination, possibly due to a shared environment (*e.g.,* local weather condition, resource availability)^24^. Therefore, studying the environmental effects on pair coordination is central to understand possible ecological constraints to the emergence of parental behaviours and their fitness consequences.

Urban environments and its comparison to natural and rural habitats provide a natural experiment to compare environmental differences in parental behaviours. The effects of urbanization on natural landscapes are numerous, from habitat fragmentation to alteration of environmental variables, *e.g.*, via heat island effects, traffic noise, and artificial light at night^26-28^. In turn, these environmental changes have profound effects at the ecosystem level, modifying resource availability and ultimately breeding behaviour and fitness of urban populations^29^. For examples, correlative and experimental studies on insectivorous songbird breeding have shown that limited and poor-quality resource availability limits provisioning behaviour and reduces reproductive success in urban habitats^29-35^. Therefore, it is likely that differences in resource distribution and abundance as a result of urbanization may lead to certain parental behaviours to be more prevalent in urban areas with fitness consequences^36,37^.

Here, we investigated whether parental coordination during the chick rearing period differs between a suburban and a rural population of house wrens, *Troglodytes aedon*. Specifically, we looked at differences in parental provisioning behaviours between the two sites and explored whether these changes were associated to differences in (i) habitat composition and (ii) arthropod (prey) abundance and how they related to reproductive success. We predicted that differences in tree and landscape composition between suburban and rural sites would be associated to differences in prey abundance, which will have effects on parental behaviours and reproductive success. Specifically, we hypothesized that the suburban area has less available prey in a more fragmented habitat that will be related to more irregular provisioning trips by the parents and lower pair coordination during offspring feeding and lower reproductive success.

**Results**

The suburban site and its rural counterpart greatly differ in terms of urbanization score and vegetation composition (table 1 and supplementary figure 1). The suburban site is characterized by a higher density of buildings and paved surfaces and by a decreased vegetation density compared to the rural site. Furthermore, coniferous trees are most abundant in the suburban site, while deciduous trees are predominant at the rural site (table 1).

Daily caterpillar biomass differed over the breeding season in interaction with site (figure 1, table 2). Post-hoc tests indicated that the difference in caterpillar abundance between sites occurred around the caterpillar peak and not at the beginning and end of the breeding season (see supplementary table 2). Furthermore, the significant interaction of site and date^2^ indicate that the curvature of the peak of caterpillar abundance differed by site*, i.e.*, the peak is more pronounced at the rural site (figure 1). In addition, the caterpillar peak occurred early in the suburban site compared to the rural counterpart (t_3.38_ = 3.39, *p* = 0.035; rural site: 156.9 ± 0.46 (mean Julian day ± SE); suburban site: 150.56 ± 1.81).

The proportion of prey delivered at the nest differed between the two sites. In rural nests, caterpillars were delivered at the nest more often than spiders and other prey types (flying insects and beetles), whereas in suburban nests other prey types were delivered more often than caterpillars and spiders (figure 2, table 3). However, rural males delivered more caterpillars and fewer spiders and other prey types compared to rural females (table 3), whereas suburban males delivered fewer caterpillars and more spiders compared to suburban females (figure 2, table 3).

Parental provisioning rates differed between sites (Cohen’s *d* = 0.91 [CI: 0.22 – 1.60]), with parents provisioning at higher rates in rural nests (figure 3a, table 4a). There was no significant effect of sex in interaction with site (*F*_1,17_ = 0.91, *p* = 0.353) or sex fitted as a single term, suggesting that the two parents had similar provisioning rates. Female parents were significantly more regular in their provisioning compared to male parents while controlling for brood size (figure 3b, table 4b), but this difference was not related to site (*F*_1,15.86_ = 0.20, *p* = 0.663 for the single effect of site, Cohen’s *d* = -0.22 [CI: -0.90 – 0.44]; *F*_1,16.56_ = 0.07, *p* = 0.800 for the interaction of sex with site). Alternation score significantly differed between suburban and rural sites (Cohen’s *d* = 1.27 [CI: 0.21 – 2.33], figure 4a, table 4c). Specifically, only parents in the rural site alternated their visits more than expected by chance (alternation score significantly differed from zero in the rural site: *t*_9_ = 4.64, *p* = 0.001; but not in the suburban site: *t*_8_ = 0.54, *p* = 0.603). In addition, the proportion of synchronized visits was higher in rural nests than in suburban nests (Cohen’s *d* = 1.86 [CI: 0.70 – 3.02]), while controlling for provisioning rate and relative proportion of parental visits by the two parents (figure 4b, table 4d). Lastly, the number of fledglings did not differ between sites (χ^2^_1_ = 1.11, *P* =0.292, Cohen’s *d* = 0.48 [CI: -0.50 – 1.46]; rural nests: 5.2 ± 0.33 (mean fledgling number ± SE); suburban nests: 4.77 ± 0.22), but rural nests fledged heavier chicks (*F*_1,16_ = 15.69, *p* = 0.001, Cohen’s *d* = 1.59 [CI: 1.12 – 2.06], figure 4c) while controlling for brood size (*F*_1,16_ = 0.03, *p* = 0.853).

**Discussion**

By comparing house wren pairs breeding in a rural and suburban site, we found that pair coordination of the provisioning differed between the sites, with parents being more coordinated in the rural environment. This difference was associated with divergent environmental conditions between sites, such as habitat fragmentation, tree composition and caterpillar availability, and rural young were fed more caterpillars than suburban young and weighed more at fledgling.

Differences in ecological conditions between rural and urban sites have an important impact on reproduction in avian species. The rural and suburban site of this study are characterized by different landscapes and composition of tree species. We found that deciduous and native tree species were predominant in the rural site, whereas the suburban site was mainly composed of coniferous species and non-native deciduous trees. This difference is likely to cause a large decrease in insect abundance^38,39^, especially for caterpillars of the *Phryganidia spp* which are more abundant on deciduous trees rather than on coniferous trees^40,41^. In this regard, similar to previous studies^30,31,37,42^ we found that caterpillar biomass was higher in the rural site compared to the suburban counterpart. Moreover, we noticed a difference in the caterpillar phenology between sites such that the peak of caterpillar production was early by *ca.* seven days in the suburban site. Early caterpillar emergence and production could be the results of urban heat island effect^28^, which is known to advance vegetation phenology^43^. In this study, however, we have not explored differences in leaf emergence phenology and insect preference for native or non-native plants between the sites and therefore we cannot draw firm conclusions about the mechanisms causing an earlier and smaller caterpillar peak in our suburban site.

Lower caterpillar biomass at the suburban site was associated with lower provisioning rates at the nest and lower proportion of caterpillar fed to the offspring by house wren parents. Differences in parental behaviours such as provisioning rate and type of food item delivered at the nest between rural and urban environments are well documented^30,37^. Some studies on passerine birds have shown either an increase in provisioning rate in urban sites^30,37^ or no difference with rural ones^32^, possibly because parents at least compensated for the lack of primary food source by bringing larger quantities of other prey types, such as spiders, flying insects, and beetles. We found that provisioning rate was lower in the suburban site even though parents provided a more variegated diet to the offspring. Discrepancies in provisioning rates at urban sites among studies could be due to different insect or food availability between the study locations or differences in prey selectivity by the parents^44,45^. We show that parents in our suburban site increased the proportion of alternative food items, which are of lower quality^46^ and may be responsible, together with lower provisioning rates, for lower offspring mass in the suburban site than in the rural one^47^. A previous study on house wrens also found nestlings weighting less at suburban sites than rural ones with authors suggesting that it may be due to lower average quality of prey^48^. We provide support for this hypothesis with similar findings of lower offspring mass in suburban areas. Lower weight at fledging is linked to lower survival, especially for migrating bird species^49^. Although urban wrens may raise similar numbers of offspring, fewer may survive past the winter. These fitness differences have implications for population dynamics in urban areas^50,51^. Interestingly, we also found that males provided more caterpillars than females, but only in the rural site. These sex differences in prey delivery suggest that parents may use different foraging strategies^52-54^ or that males are better foragers of high-quality prey. Alternatively, females may be more responsive to begging behaviours of the offspring and may favour consistency over quality in their provisioning^55^. In addition, we found that at both sites, males have less regular feeding intervals compared to females despite having similar provisioning rates. This could be explained by males foraging in different locations more often than females^56^. Further studies on sex differences between rural and urban populations are needed to better understand whether urbanization poses different selective pressures on the sexes. We expected more irregular provisioning trips at the suburban site, possibly due to a more fragmented habitat, but we did not notice a difference with the rural counterpart. This could be explained by suburban parents foraging on the same number of patches but choosing less profitable prey items to maintain regular feedings to the offspring. Radio tracking studies are needed to better investigate the relationship between visit patterns at the nest (provisioning rate and regularity) and foraging behaviour between urbanized and rural environments.

Pair coordination of the provisioning differed between sites, with higher alternation and synchrony of nest visits in the rural site compared to the suburban one. Why do rural parents have more coordinated provisioning? Four non-mutually exclusive scenarios are possible. First, spatial heterogeneity (habitat fragmentation), lower food availability or human disturbance in the urban environment may decrease coordinated behaviours because parents may need to forage in different locations^24^ or further away from the nest^57^. Empirical evidence in zebra finches *Taeniopygia guttata* showed that parents foraging independently from each other at different foraging areas decreased synchrony of nest visits^16^. Urban areas, such as our suburban site, may thus induce parents to forage independently from each other more often, resulting in lower coordination of their nest visits. A recent radio-tracking study on blue tits *Cyanistes caeruleus* showed differences in foraging behaviour between populations at an urban and rural site in that provisioning trips occur on average further from the nest for parents nesting in an urban site compared to a forest site^57^. However, to the best of our knowledge, it is not known whether urban environments also affect other aspect of the foraging behaviour, such as the number of foraging locations or the coordination between the parents (whether parents forage together or independently from each other).Further telemetry studies with both parents tracked simultaneously are necessary to shed lights on space use and pair coordination of foraging parents in urban areas. Second, urban and rural environments may have different predation risks affecting parental provisioning^58,59^. A review on predation risk in urban areas reported a consistent decrease of predation rate along rural-to-urban gradients on several continents^59^ (note however that this effect seems to be only valid for natural nests)^60^. Synchrony of the nest visits has been considered as an antipredator behaviour, which minimizes conspicuous activity at the nest^15,17,18^. Therefore, parental coordination might be higher in rural areas in response to higher predation risk. Third, there could be an age difference between individuals nesting in rural and urban sites leading to more coordinated care associated with increased experience^61,62^. Studies have suggested an age-specific settlement and an habitat-dependent survival in urban or novel sites^63-65^. For example, if the individuals settling in urban areas are younger and less experienced, this age-related distribution could explain our results. Lastly, urban noise disrupts communication and acoustic coordination^66-68^. There is good evidence that songbirds sing at different frequencies in the presence of urban noise pollution^69^. Therefore, urban areas may disrupt vocal communication, both between parents and between parents and offspring that facilitate coordinated behaviours^70^. These four scenarios could be experimentally tested using existing rural and urban populations. As such, urbanization research could provide valuable insights on the extent to which pair coordination results from or is constrained by environment composition (*e.g.* habitat fragmentation, predation risk) or by pair characteristics and behaviour (*e.g.* experience and vocal communication).

Our findings that urbanization is associated with reduced parental coordination have important potential fitness consequences for offspring. First, less alternating parents could monitor and respond to their partner’s activity less often, and in a turn-taking framework, they are expected to invest at a rate which is lower than their maximum^13,71^. Therefore, urbanization could in theory strengthen sexual conflict between the parents with negative consequences for offspring growth and fitness. In this respect, we found that suburban pairs fledged lighter chicks. However, it is notoriously difficult to assess the impact of reduced alternation alone on offspring fitness in correlative studies^72,73^, especially in situations in which fledgling weight is also likely to be function of provisioning rate alone, food availability and habitat composition such as in this study. Experimental manipulations of one parent’s investment (*e.g*. via handicapping or selective playback experiment) with a concomitant food supplementation (to eliminate environmental constraints on parental care) might be a reasonable approach to investigate the effect of pair coordination alone on offspring fitness. Second, reduced synchrony of the nest visits in urban environments could also strengthen offspring conflict over resource allocation. It has been shown that synchronized feedings at the nest are related to more equal division of food between offspring in a cooperative breeding bird^19^. However, in this study we did not investigate food partitioning between the offspring and cannot explore differences in food allocation between rural and suburban nests.

Our study indicates that, despite a relatively small sample size of nests, different levels of parental coordination exist between pairs breeding in a rural environment compared to a suburban one and discusses how these differences could be driven by diverse ecological and environmental conditions. We promote further studies on replicated urban and rural sites to assess the generality of our findings. Furthermore, we emphasize that comparing populations breeding along an urbanization gradient represents a valuable tool to study environmental effects on parental behaviours and advocate new studies on the behavioural mechanisms driving parental coordination.

**Methods**

*Characterization of suburban and rural sites*

We conducted our study from May to July 2018 at one suburban and one rural site in Reno, Nevada, USA (table 1), which were set up with artificial nest boxes since 2016. The distance between the two sites is 10.8 km. Our suburban site was located near Caughlin Ranch, which is a suburban park (supplementary figure 1). This park is located within a suburban neighbourhood with paved walkaways and artificial ponds that fragment the green spaces in pockets of vegetation. Our rural site was the University of Nevada, Reno, Agricultural Experiment Station, which is a university owned agricultural farm with ~1000 acres of farmland and pastures (supplementary figure 1). The nest-box population at the rural site was set up in a riparian habitat along the Truckee River, in which vegetation is condensed in tree clusters along two lines. These two sites differed in terms of urbanization score and vegetation composition (table 1). Urbanization score was estimated as the land use of each study site, using the validated method described by Seress, et al. ^74^. This approach divides an aerial image of the 1 km^2^ area around each study site into 100 × 100 m cells and then scores the abundance of vegetation, buildings, and paved surfaces, such as roads and parking lots, in each cell. The suburban site has a higher urbanization score than the rural counterpart by having more cells with increased building density and paved surfaces and decreased vegetation density (table 1). Furthermore, a complete tree census, where we marked individual trees with GPS points (handheld Garmin GPSMAP 62st), indicates that vegetation composition differs between the two sites, in that coniferous trees are predominant in the suburban site, while the rural site is mainly composed by deciduous trees (table 1).

House wrens are secondary cavity nesters that readily make use of manmade nest-boxes. They prefer open woodland habitat, rarely nesting more than 30 m from woody vegetation but also avoiding dense wooded nest sites^75^. Both males and females feed offspring with a diverse diet of invertebrates, with adult lepidoptera and caterpillars (49%) making up the bulk of the food items brought to offspring and spiders (32%) as a second choice^76,77^.

*Estimation of caterpillar abundance*

From mid-April to mid-July, we collected caterpillar frass (n=80) under oak trees at both sites using 1 x 1 m^2^ cheesecloths (n=4 per site per 10 sampling dates). We emptied all nets every week (7.1 ± 0.15 (mean days between frass sample collection ± SE)) at the same time (0800-0900h). We dried frass for 2 hours in a 60°C oven and then picked out the frass under a dissecting microscope (40x zoom). We weighed the dried mass to the nearest 0.0001g. Caterpillar biomass was estimated after correcting for temperature using the methods described in Welbers, et al. ^78^.

*Collection of provisioning data*

From the beginning of May, we monitored house wren nests every week to determine the onset of egg laying and incubation at both field sites. We then checked active nests daily from the day before the predicted hatching to determine the exact hatch date (day 0). At day 8 of chick age, we caught the parents at the nest and banded them with a unique combination of coloured rings. At day 10, we observed parental behaviour for one hour in the morning for 19 nests (ten located in the rural site and nine in the suburban site). At day 15, we measured chick weights at fledging (between 0800-1000h). For this study we only used unmanipulated nests which were not part of a cross-foster experiment^47^. One-hour behavioural observations were carried out by JQO with a binocular while sitting in the open, approximately 30m from the nest. Observations started 30 min after approaching the nest to habituate house wren parents to our presence. Nests in the rural and suburban site were observed around the same period during the breeding season and time of the day (mean Julian date and time of the observations does not differ between sites: *F*_1,17_ = 0.01, *p* = 0.94 for Julian date; *F*_1,17_ = 0.40, *p* = 0.53 for observation time, see supplementary table 1). For each parental visit at the nest, we noted: i) the sex of the visiting parent (identifiable by the ring colour combination), ii) the time that the bird entered the nest-box (to the nearest second), and iii) the type of delivered prey divided into three categories: caterpillars (lepidopteran larvae), spiders and others (flying insects and beetles). Unidentifiable items represented 1 % (n= 4) of 293 total provisioning trips and were excluded from the analyses. A pilot study with behavioural observations and video recordings of house wren nests in the previous year indicated a 98% accuracy of behavioural observations in identifying prey items for each visit. All of the data were collected under the appropriate state and federal permits and approved IACUC protocols.

*Calculation of alternation and synchrony of the nest visits*

From the sequence of nest visits, we calculated pair coordination, measured as alternation and synchrony of the nest visits. We defined alternated visits as visits of one individual that followed a visit of its mate. For the calculation of alternation from a sequence of nest visits (e.g. MFFMFMFMM), visits can occur at any time, and by either parent, after the previous one. We expected different amounts of alternation to arise by chance in a sequence of visits depending on the proportion of visits by the two parents. In situations in which, for instance, one parent makes either all or none of the visits in a sequence, no alternated visits can occur. Conversely, when parents feed the offspring at similar rates, the proportion of alternated visits we expected by chance increases. To account for this effect, we used an alternation score to measure the deviation of the observed amount of alternation from that expected given the relative contributions (provisioning rates) of the two parents using the following formula from Baldan, et al. ^21^ :

$$\text{Alternation score}=\log\left( \frac{\text{Observed no. of alternated visits}}{\text{Observed no. of nonalternated visits}} \right)-\log\left( \frac{\text{Expected no. of alternated visits}}{\text{Expected no. of nonalternated visits}} \right)$$

An alternation score of zero represents the amount of alternation expected by chance, a value of less than zero indicates that the observed alternation is lower than expected by chance, whereas a value of greater than zero indicates that the observed alternation is greater than expected by chance. See Baldan, et al. ^21^ for a detailed explanation of the calculation of the alternation score.

We also calculated the proportion of synchronized visits as the number of synchronized visits over the total number of visits. Synchronized visits were defined as a pair of visits (one by each parent), which occurred within one minute of each other. Like previous studies^17,79^, we used a one-minute window to calculate synchrony to minimize the risk that synchronized visits could occur by chance (see supplementary figure 2 for distribution of the time intervals between two consecutive visits). In our dataset, males and females in the rural site visited the nest on average 10.2 and 9.9 times per hour respectively, whereas in the suburban site they visited on average 5.6 and 7.1 times per hour respectively. If parents were visiting the nest independently from each other, we would expect that parental visits occurring by chance within one minute of each other would be less than 3% [(10.2 male visit rate / 60 seconds) X (9.9 female visit rate / 60 seconds)] in the rural site and 1.1% [(5.6 male visit rate / 60 seconds) X (7.1 female visit rate / 60 seconds)] in the suburban site.

*Statistical analyses*

To investigate differences in caterpillar abundance between the two sites, we used two approaches. First, we used a linear mixed model (LMM) to test whether daily caterpillar biomass differed between sites and over the breeding season. We fitted ‘daily caterpillar biomass’ as response variable, ‘date’ and its quadratic term in interaction with ‘site’ (suburban and rural) as fixed effects and ‘frass net ID’ as a random effect to account for repeated measures. We fitted this LMM with the lme function (nlme package^80^), allowing heterogeneous variances (heteroscedasticity) between the two sites (varIdent argument within the lme function). Second, we investigated whether the timing of the caterpillar peak differed between sites. For each frass net, we estimated the date of maximum peak using the cardidate R package^81^. This methodology fits curves to environmental time series using Weibull-Functions and estimates the beginning, maximum and end dates of ecological processes^81^, such as the phenology in caterpillar biomass. We then compared the time of caterpillar peaks between sites (expressed as Julian date) using a two-samples t-test. We investigated whether the proportion of prey type delivered at the nest differed between sites. We fitted multinomial logit models^82^ to model the proportion of prey delivered (divided into three categories: caterpillar, spiders and others) in relation to ‘site’ and ‘sex’ and their interaction. ‘Nest ID’ was included in the model as random effect, and the prey counts (from which the proportions are derived) were included as weight. Multinomial logit models were fitted using the function *mblogit* in the *mclogit* package^83^.

To explore whether parental behaviour differed between suburban and rural nests, we first explored parental provisioning rates and regularity. For each parent we calculated individual provisioning rate as the number of provisioning trips at the nest per hour. We fitted a LMM with ‘individual provisioning rate’ as the response variable, ‘site’, ‘sex’, and their interaction as fixed effects, while controlling for ‘brood size.’ ‘Nest ID’ was included in the model as the random effect. Individual provisioning rate was log transformed to normalize the model residuals. We then explored male and female regularity of the inter-visit intervals (time intervals between two consecutive visits by the same parent, henceforward abbreviated to IVIs). Similarly to a previous study^21^, we expressed regularity as the coefficient of variation (CV) of the IVIs (i.e. standard deviation/mean). Low CV values indicate higher regularity of the IVIs (lower standard deviation compared to the mean), whereas high CV values indicate lower regularity of the IVIs (higher standard deviation compared to the mean value). Here we fitted a LMM with ‘CV of the IVIs’ as the response variable, ‘site’, ‘sex’, their interaction and brood size as fixed effects, and ‘Nest ID’ as the random effect. We then investigated whether alternation of nest visits differed between suburban and rural nests. Here we fitted a linear model with ‘alternation score’ as the response variable, ‘site’ as factor and ‘brood size’ as covariates. Furthermore, we explored whether synchrony varied between sites by fitting a generalized linear model (family quasi-binomial to control for overdispersion; overdispersion parameter= 2.02) with proportion of synchronized visits as the response variable, ‘site’ as a fixed effect and ‘brood size’ as covariate. In this analysis, we also included ‘total number of visits’ and ‘proportion of male visits’ as covariates, as we expected i) the amount of synchronized visits to decrease as the difference in proportion of feeds by the two parents increases and ii) synchrony increases at higher feeding rates, as it increases the chance that two visits can occur within one minute from each other. Lastly, we tested whether fledging success (number of young fledged) differed between rural and suburban nests. Here we fitted i) a generalized linear model (family quasi-Poisson to control for dispersed data; overdispersion parameter= 0.15) for fledgling number, and ii) a LMM for individual chick weight.

All the statistical analyses were performed in R environment (version 3.6; R Development Core Team, 2017). All mixed models were performed with the *lmer* function in the *lme4* package^84^. For all models with interaction terms, we first tested whether the interactions were significant. If the interaction terms were non-significant, they were removed from the final model. Cohen's *d* and its 95% confidence interval were calculated as a measure of effect size for the variable *site* in our models^85,86^. Post-hoc tests were carried out using the *emmeans* function in the *emmeans* package^87^. Significance was taken at α = 0.05 and all model assumptions were met.

**References**

1 Royle, N. J., Smiseth, P. T. & Kölliker, M. *The evolution of parental care*. (Oxford University Press, 2012).

2 Williams, G. C. Natural Selection, the Costs of Reproduction, and a Refinement of Lack's Principle. *The American Naturalist* **100**, 687-690 (1966).

3 Trivers, R. L. in *Sexual Selection and the Descent of Man* 136-179 (Aldine Press Chicago, 1972).

4 Lessells, C. M. in *The Evolution of Parental Care* (Oxford univeristy Press, 2012).

5 Houston, A. I., Székely, T. & McNamara, J. M. Conflict between parents over care. *Trends in Ecology & Evolution* **20**, 33-38 (2005).

6 Lessells, C. M. The evolutionary outcome of sexual conflict. *Philosophical Transactions of the Royal Society B-Biological Sciences* **361**, 301-317 (2006).

7 Houston, A. I. & Davies, N. B. The evolution of cooperation and life history in the dunnock, *Prunella modularis*. *Behavioural Ecology: Ecological Consequences of Adaptive Behaviour*, 471-487 (1985).

8 McNamara, J. M., Gasson, C. E. & Houston, A. I. Incorporating rules for responding into evolutionary games. *Nature* **401**, 368-371 (1999).

9 McNamara, J. M., Houston, A. I., Barta, Z. & Osorno, J. L. Should young ever be better off with one parent than with two? *Behavioral Ecology* **14**, 301-310 (2003).

10 Lessells, C. M. & McNamara, J. M. Sexual conflict over parental investment in repeated bouts: negotiation reduces overall care. *Proceedings of the Royal Society B-Biological Sciences* **279**, 1506-1514 (2012).

11 Johnstone, R. A. & Hinde, C. A. Negotiation over offspring care - how should parents respond to each other's efforts? *Behavioral Ecology* **17**, 818-827 (2006).

12 Royle, N. J., Hartley, I. R. & Parker, G. A. Sexual conflict reduces offspring fitness in zebra finches. *Nature* **416**, 733-736 (2002).

13 Johnstone, R. A. *et al.* Reciprocity and conditional cooperation between great tit parents. *Behavioral Ecology* **25**, 216-222 (2014).

14 Savage, J. L., Browning, L. E., Manica, A., Russell, A. F. & Johnstone, R. A. Turn-taking in cooperative offspring care: by-product of individual provisioning behavior or active response rule? *Behavioral Ecology and Sociobiology* **71**, 162 (2017).

15 Raihani, N. J., Nelson-Flower, M. J., Moyes, K., Browning, L. E. & Ridley, A. R. Synchronous provisioning increases brood survival in cooperatively breeding pied babblers. *Journal of Animal Ecology* **79**, 44-52 (2010).

16 Mariette, M. M. & Griffith, C. S. The Adaptive Significance of Provisioning and Foraging Coordination between Breeding Partners. *The American Naturalist* **185**, 270-280 (2015).

17 Bebbington, K. & Hatchwell, B. J. Coordinated parental provisioning is related to feeding rate and reproductive success in a songbird. *Behavioral Ecology* **27**, 652–659 (2016).

18 Leniowski, K. & Węgrzyn, E. Synchronisation of parental behaviours reduces the risk of nest predation in a socially monogamous passerine bird. *Scientific Reports* **8**, 7385 (2018).

19 Shen, S. F., Chen, H. C., Vehrencamp, S. L. & Yuan, H. W. Group provisioning limits sharing conflict among nestlings in joint-nesting Taiwan yuhinas. *Biology Letters* **6**, 318-321 (2010).

20 Savage, J. L. & Hinde, C. A. What Can We Quantify About Carer Behavior? *Frontiers in Ecology and Evolution* **7**, doi:10.3389/fevo.2019.00418 (2019).

21 Baldan, D., Curk, T., Hinde, C. A. & Lessells, C. M. Alternation of nest visits varies with experimentally manipulated workload in brood-provisioning great tits. *Animal Behaviour* **156**, 139-146, doi:https://doi.org/10.1016/j.anbehav.2019.08.004 (2019).

22 Griffioen, M., Müller, W. & Iserbyt, A. A fixed agreement—consequences of brood size manipulation on alternation in blue tits. *PeerJ* **7**, e6826, doi:10.7717/peerj.6826 (2019).

23 Iserbyt, A., Fresneau, N., Kortenhoff, T., Eens, M. & Muller, W. Decreasing parental task specialization promotes conditional cooperation. *Scientific Reports* **7** (2017).

24 Baldan, D., Hinde, C. A. & Lessells, C. M. Turn-taking between provisioning parents: partitioning alternation. *Frontiers in Ecology and Evolution* **7**, doi:10.3389/fevo.2019.00448 (2019).

25 Lejeune, L. *et al.* Environmental Effects on Parental Care Visitation Patterns in Blue Tits Cyanistes caeruleus. *Frontiers in Ecology and Evolution* **7**, doi:10.3389/fevo.2019.00356 (2019).

26 Longcore, T. & Rich, C. Ecological light pollution. *Frontiers in Ecology and the Environment* **2**, 191-198, doi:10.1890/1540-9295(2004)002[0191:Elp]2.0.Co;2 (2004).

27 Warren, P. S., Katti, M., Ermann, M. & Brazel, A. Urban bioacoustics: it's not just noise. *Animal Behaviour* **71**, 491-502, doi:https://doi.org/10.1016/j.anbehav.2005.07.014 (2006).

28 McCarthy, M. P., Best, M. J. & Betts, R. A. Climate change in cities due to global warming and urban effects. *Geophysical Research Letters* **37**, doi:10.1029/2010gl042845 (2010).

29 CHAMBERLAIN, D. E. *et al.* Avian productivity in urban landscapes: a review and meta-analysis. *Ibis* **151**, 1-18, doi:10.1111/j.1474-919X.2008.00899.x (2009).

30 Pollock, C. J., Capilla-Lasheras, P., McGill, R. A. R., Helm, B. & Dominoni, D. M. Integrated behavioural and stable isotope data reveal altered diet linked to low breeding success in urban-dwelling blue tits (Cyanistes caeruleus). *Scientific Reports* **7**, 5014, doi:10.1038/s41598-017-04575-y (2017).

31 Seress, G. *et al.* Impact of urbanization on abundance and phenology of caterpillars and consequences for breeding in an insectivorous bird. *Ecological Applications* **28**, 1143-1156, doi:10.1002/eap.1730 (2018).

32 Seress, G., Sándor, K., Evans, K. L. & Liker, A. Food availability limits avian reproduction in the city: An experimental study on great tits Parus major. *Journal of Animal Ecology* **00**, 1-11, doi:10.1111/1365-2656.13211 (2020).

33 Wilkin, T. A., King, L. E. & Sheldon, B. C. Habitat quality, nestling diet, and provisioning behaviour in great tits *Parus major*. *Journal of Avian Biology* **40**, 135-145, doi:10.1111/j.1600-048X.2009.04362.x (2009).

34 Peach, W. J., Mallord, J. W., Ockendon, N., Orsman, C. J. & Haines, W. G. Depleted suburban house sparrow Passer domesticus population not limited by food availability. *Urban Ecosystems* **21**, 1053-1065, doi:10.1007/s11252-018-0784-4 (2018).

35 Schoech, S. J. *et al.* Food supplementation: A tool to increase reproductive output? A case study in the threatened Florida Scrub-Jay. *Biological Conservation* **141**, 162-173, doi:https://doi.org/10.1016/j.biocon.2007.09.009 (2008).

36 Sol, D., Lapiedra, O. & González-Lagos, C. Behavioural adjustments for a life in the city. *Animal Behaviour* **85**, 1101-1112, doi:https://doi.org/10.1016/j.anbehav.2013.01.023 (2013).

37 Isaksson, C. & Andersson, S. Carotenoid diet and nestling provisioning in urban and rural great tits Parus major. *Journal of Avian Biology* **38**, 564-572, doi:10.1111/j.2007.0908-8857.04030.x (2007).

38 New, T. R. *Insect Conservation and Urban Environments*. (Springer International Publishing, 2015).

39 Helden, A., Stamp, G. & Leather, S. Urban biodiversity: comparison of insect assemblages on native and non-native trees. *Urban Ecosystems* **15**, 611-624, doi:10.1007/s11252-012-0231-x (2012).

40 Tallamy, D. W. & Shropshire, K. J. Ranking Lepidopteran Use of Native versus Introduced Plants. *Conservation Biology* **23**, 941-947 (2009).

41 Burghardt, K. T., Tallamy, D. W., Philips, C. & Shropshire, K. J. Non-native plants reduce abundance, richness, and host specialization in lepidopteran communities. *Ecosphere* **1**, art11, doi:10.1890/es10-00032.1 (2010).

42 Marciniak, B., Nadolski, J., Nowakowska, M., Loga, B. & Bańbura, J. Habitat and Annual Variation in Arthropod Abundance Affects Blue Tit <i>Cyanistes caeruleus</i> Reproduction. *Acta Ornithologica* **42**, 53-62, 10 (2007).

43 Neil, K. & Wu, J. Effects of urbanization on plant flowering phenology: A review. *Urban Ecosystems* **9**, 243-257, doi:10.1007/s11252-006-9354-2 (2006).

44 Lessells, C. M. & Stephens, D. W. Central place foraging: single-prey loaders again. *Animal Behaviour* **31**, 238-243 (1983).

45 Orians, G. H. & Pearson, N. E. On the theory of central place foraging. *Analysis of ecological systems. Ohio State University Press, Columbus*, 155-177 (1979).

46 Arnold, K. E., Ramsay, S. L., Henderson, L. & Larcombe, S. D. Seasonal variation in diet quality: antioxidants, invertebrates and blue tits *Cyanistes caeruleus*. *Biological Journal of the Linnean Society* **99**, 708-717, doi:10.1111/j.1095-8312.2010.01377.x (2010).

47 Ouyang, J. Q., Baldan, D., Munguia, C. & Davies, S. Genetic inheritance and environment determine endocrine plasticity to urban living. *Proceedings of the Royal Society B: Biological Sciences* **286**, 20191215, doi:doi:10.1098/rspb.2019.1215 (2019).

48 Newhouse, M. J., Marra, P. P. & Johnson, L. S. Reproductive success of house wrens in suburban and rural landscapes. *The Wilson Journal of Ornithology* **120**, 99-104 (2008).

49 Potti, J., Dávila, J. A., Tella, J. L., Frías, Ó. & Villar, S. Gender and viability selection on morphology in fledgling pied flycatchers. *Molecular Ecology* **11**, 1317-1326, doi:10.1046/j.1365-294X.2002.01545.x (2002).

50 Balogh, A. L., Ryder, T. B. & Marra, P. P. Population demography of Gray Catbirds in the suburban matrix: sources, sinks and domestic cats. *Journal of Ornithology* **152**, 717-726, doi:10.1007/s10336-011-0648-7 (2011).

51 Stillfried, M. *et al.* Do cities represent sources, sinks or isolated islands for urban wild boar population structure? *Journal of Applied Ecology* **54**, 272-281, doi:doi:10.1111/1365-2664.12756 (2017).

52 Holmes, R. T. Foraging Patterns of Forest Birds: Male-Female Differences. *The Wilson Bulletin* **98**, 196-213 (1986).

53 Chaves, F. G., Vecchi, M. B. & Alves, M. A. S. Intersexual differences in the foraging behavior of Formicivora littoralis (Thamnophilidae), an endangered Neotropical bird. *Studies on Neotropical Fauna and Environment* **52**, 179-186, doi:10.1080/01650521.2017.1335275 (2017).

54 Mänd, R., Rasmann, E. & Mägi, M. When a male changes his ways: sex differences in feeding behavior in the pied flycatcher. *Behavioral Ecology* **24**, 853-858, doi:10.1093/beheco/art025 (2013).

55 Kölliker, M., Brinkhof, M. W. G., Heeb, P., Fitze, P. S. & Richner, H. The quantitative genetic basis of offspring solicitation and parental response in a passerine bird with biparental care. *Proceedings of the Royal Society of London. Series B: Biological Sciences* **267**, 2127-2132 (2000).

56 Naef-Daenzer, B. Patch time allocation and patch sampling by foraging great and blue tits. *Animal Behaviour* **59**, 989-999 (2000).

57 Jarrett, C., Powell, L. L., McDevitt, H., Helm, B. & Welch, A. J. Bitter fruits of hard labour: diet metabarcoding and telemetry reveal that urban songbirds travel further for lower-quality food. *Oecologia* **193**, 377-388, doi:10.1007/s00442-020-04678-w (2020).

58 Gering, J. C. & Blair, R. B. Predation on artificial bird nests along an urban gradient: predatory risk or relaxation in urban environments? *Ecography* **22**, 532-541, doi:10.1111/j.1600-0587.1999.tb01283.x (1999).

59 Fischer, J. D., Cleeton, S. H., Lyons, T. P. & Miller, J. R. Urbanization and the Predation Paradox: The Role of Trophic Dynamics in Structuring Vertebrate Communities. *BioScience* **62**, 809-818, doi:10.1525/bio.2012.62.9.6 (2012).

60 Vincze, E. *et al.* Does Urbanization Affect Predation of Bird Nests? A Meta-Analysis. *Frontiers in Ecology and Evolution* **5**, doi:10.3389/fevo.2017.00029 (2017).

61 Griggio, M. & Hoi, H. An experiment on the function of the long-term pair bond period in the socially monogamous bearded reedling. *Animal Behaviour* **82**, 1329-1335, doi:https://doi.org/10.1016/j.anbehav.2011.09.016 (2011).

62 Griffith, S. C. Cooperation and Coordination in Socially Monogamous Birds: Moving Away From a Focus on Sexual Conflict. *Frontiers in Ecology and Evolution* **7**, doi:10.3389/fevo.2019.00455 (2019).

63 Alberti, M. Eco-evolutionary dynamics in an urbanizing planet. *Trends Ecol Evol* **30**, 114-126, doi:10.1016/j.tree.2014.11.007 (2015).

64 Liebl, A. L. & Martin, L. B. Exploratory behaviour and stressor hyper-responsiveness facilitate range expansion of an introduced songbird. *Proc Biol Sci* **279**, 4375-4381, doi:10.1098/rspb.2012.1606 (2012).

65 Sepp, T., McGraw, K. J., Kaasik, A. & Giraudeau, M. A review of urban impacts on avian life-history evolution: Does city living lead to slower pace of life? *Global Change Biology* **24**, 1452-1469, doi:10.1111/gcb.13969 (2018).

66 Patricelli, G. L. & Blickley, J. L. Avian Communication in Urban Noise: Causes and Consequences of Vocal Adjustment. *The Auk* **123**, 639-649, doi:10.1093/auk/123.3.639 (2006).

67 Grabarczyk, E. E. & Gill, S. A. Anthropogenic noise affects male house wren response to but not detection of territorial intruders. *PLOS ONE* **14**, e0220576, doi:10.1371/journal.pone.0220576 (2019).

68 Schroeder, J., Nakagawa, S., Cleasby, I. R. & Burke, T. Passerine Birds Breeding under Chronic Noise Experience Reduced Fitness. *PLOS ONE* **7**, e39200, doi:10.1371/journal.pone.0039200 (2012).

69 Halfwerk, W. *et al.* Low-frequency songs lose their potency in noisy urban conditions. *Proceedings of the National Academy of Sciences* **108**, 14549-14554, doi:10.1073/pnas.1109091108 (2011).

70 Mariette, M. M. Acoustic Cooperation: Acoustic Communication Regulates Conflict and Cooperation Within the Family. *Frontiers in Ecology and Evolution* **7**, doi:10.3389/fevo.2019.00445 (2019).

71 Johnstone, R. A. & Savage, J. L. Conditional Cooperation and Turn-Taking in Parental Care. *Frontiers in Ecology and Evolution* **7**, doi:10.3389/fevo.2019.00335 (2019).

72 Ihle, M., Pick, J. L., Winney, I. S., Nakagawa, S. & Burke, T. Measuring Up to Reality: Null Models and Analysis Simulations to Study Parental Coordination Over Provisioning Offspring. *Frontiers in Ecology and Evolution* **7**, doi:10.3389/fevo.2019.00142 (2019).

73 Ihle, M. *et al.* Rearing Success Does Not Improve With Apparent Pair Coordination in Offspring Provisioning. *Frontiers in Ecology and Evolution* **7**, doi:10.3389/fevo.2019.00405 (2019).

74 Seress, G., Lipovits, A., Bokony, V. & Czuni, L. Quantifying the urban gradient: A practical method for broad measurements. *Landscape and Urban Planning* **131**, 42-50, doi:10.1016/j.landurbplan.2014.07.010 (2014).

75 Johnson, L. S. in *The Birds of North America* (ed Editor A. F. Poole) (2014).

76 PEARSE, A. T., CAVITT, J. F. & CULLY, J. F. EFFECTS OF FOOD SUPPLEMENTATION ON FEMALE NEST ATTENTIVENESS AND INCUBATION MATE FEEDING IN TWO SYMPATRIC WREN SPECIES. *The Wilson Bulletin* **116**, 23-30, 28 (2004).

77 Greenewalt, C. H. & Jones, F. M. Photographic Studies of the Feeding of Nestling House Wrens. *Proceedings of the American Philosophical Society* **99**, 200-204 (1955).

78 Welbers, A. A. M. H. *et al.* Artificial Light at Night Reduces Daily Energy Expenditure in Breeding Great Tits (Parus major). *Frontiers in Ecology and Evolution* **5**, doi:10.3389/fevo.2017.00055 (2017).

79 Baldan, D. & Griggio, M. Pair coordination is related to later brood desertion in a provisioning songbird. *Animal Behaviour* **156**, 147-152, doi:https://doi.org/10.1016/j.anbehav.2019.08.002 (2019).

80 Pinheiro J, Bates D, DebRoy S, Sarkar D & Team, R. C. nlme: Linear and Nonlinear Mixed Effects Models. (2019).

81 Rolinski, S., Horn, H., Petzoldt, T. & Paul, L. Identifying cardinal dates in phytoplankton time series to enable the analysis of long-term trends. *Oecologia* **153**, 997-1008 (2007).

82 Douma, J. C. & Weedon, J. T. Analysing continuous proportions in ecology and evolution: A practical introduction to beta and Dirichlet regression. *Methods in Ecology and Evolution* **10**, 1412-1430, doi:10.1111/2041-210x.13234 (2019).

83 Martin, E. mclogit: Multinomial Logit Models, with or without Random Effects or Overdispersion. (2020).

84 Bates, D., Mächler, M., Bolker, B. & Walker, S. Fitting linear mixed-effects models using lme4. *Journal of Statistical Software* **67**, 48 (2015).

85 Cohen, J. *Statistical power analysis for the behavioral sciences*. (Lawrence Erlbaum Associates, Hillsdale, NJ, 1988).

86 Lakens, D. Calculating and reporting effect sizes to facilitate cumulative science: a practical primer for t-tests and ANOVAs. *Frontiers in Psychology* **4**, doi:10.3389/fpsyg.2013.00863 (2013).

87 Lenth, R. emmeans: Estimated Marginal Means, aka Least-Squares Means. (2020).

**Acknowledgements**

We thank the editor and two anonymous reviewers for helpful comments. We thank the Caughlin Ranch HOA, especially Lori Strong for field site accessibility. We are grateful to Jia-Yi Cen, Ryan Fung, Kristiana Hodach, Kimberlee Kiep, Michelle Schilling, and Jacquelynn Tran for assistance in the field, and to Martin Elff for statistical advice on the multinomial models. JQO is funded by the National Science Foundation (OIA-1738594) and the National Institute of Health (P20 GM103650).

**Author contributions**

D.B. and J.Q.O. conceived the study. J.Q.O. collected the data. D.B. performed the statistical analyses. D.B. and J.Q.O. wrote the paper.

**Competing interests**

The authors declare no competing interests.

**Data Availability**

The datasets analysed during the current study are available from the corresponding author on reasonable request.

**Figure legends**

**Figure 1.** Daily caterpillar biomass in the rural and suburban site (caterpillar frasses n=80) between the 12^th^ of May (Julian date 131) and the 16^th^ of July (Julian date 196). Shown are means ± SE for the rural (circles and solid line) and suburban (triangles and dashed line) sites. Asterisks represent significant differences in caterpillar biomass on a specific sampling date (* = *P* ≤ 0.05, ** = *P* ≤ 0.01, *** = *P* ≤ 0.001). Only significant comparisons are shown.

**Figure 2.** Boxplots for the proportion of prey delivered at the nest by male and female parents at the rural (a) and suburban (b) site. Preys (n= 289) were divided into three groups: caterpillars, spiders and others (flying insects and beetles).

**Figure 3.** Provisioning rates (a) and regularity of the IVIs (expressed as CV of the IVIs) (b) for male and female parents at the rural and suburban site (rural pairs n=10, suburban pairs n=9). Higher values of CV of the IVIs represent more irregular feeding intervals, whereas lower values represent more regular feedings. Shown are means ± SE.

**Figure 4.** Alternation score (a), proportion of synchronized visits (b) and fledgling weight (c) in the rural and suburban nests. An alternation score of zero (dashed line) in (a) represents the amount of alternation expected by chance, assuming the probability of a nest visit by a parent is constant with respect to time. Mean ± SE are given.

**Table 1.** Location, land use estimates, urbanization score, vegetation composition and main tree species (top 3 species) for the two study sites sampled.

|  | Site name | Latitude (N) | Longitude (W) | | Cells with high building density | Cells with high vegetation density | | Cells with paved surfaces | Mean building density | Mean vegetation density | Urbanization score | Vegetation composition | Main tree species |
| --- | --- | --- | --- | --- | --- | --- | --- | --- | --- | --- | --- | --- | --- |
| Suburban | Caughlin ranch | 39°30’03” | 119°51’ 44” | 21 | | | 59 | 32 | 0.82 | 1.59 | 2.24 | 33% deciduous trees (n: 68); 67% coniferous trees (n: 138) | Red Pine (*Pinus resinosa*) 41% (n=84);  Ponderosa pine (*Pinus ponderosa*) 16% (n=34);  Siberian Elm (*Ulmus pumila*) 9% (n=18) |
| Rural | Agricultural Experiment Station, University of Nevada, Reno | 39°30’46” | 119°44’13” | 10 | | | 88 | 10 | 0.24 | 1.87 | -2.24 | 81% deciduous trees (n: 145); 19% coniferous trees (n: 34) | White mulberry (*Morus alba*) 23% (n=41);  Black cottonwood (*Populus trichocarpa*) 18% (n=33);  Rocky mountain Juniper (*Juniperus scopulorum*) 12% (n=21) |

**Table 2.** Type II Anova table of the linear mixed model (LMM) model estimating daily caterpillar biomass in relation to Julian date and site. ‘Frass net ID’ was included as random effect. Significant p-values are shown in bold.

| Variable | *χ*^2^ | *df* | *p*-value |
| --- | --- | --- | --- |
| *LMM for daily* caterpillar biomass |  |  |  |
| Date | 5.79 | 1 | **0.016** |
| Date^2^ | 6.30 | 1 | **0.012** |
| Site | 11.46 | 1 | **<0.001** |
| Date × Site | 4.35 | 1 | **0.037** |
| Date^2^ × Site | 4.63 | 1 | **0.031** |

**Table 3.** Summary table of the multinomial logistic model investigating the difference in proportion of prey item delivered at the nest (caterpillars, spiders and others) in relation to site and parental sex**.** ‘Caterpillars’ was used as reference category in the model. Significant p-values are shown in bold.

| Variable |  |  | Estimate | *SE* | *z-score* | *p*-value |
| --- | --- | --- | --- | --- | --- | --- |
| Comparison between Insects and Caterpillars |  |  |  |  |  |  |
| (Intercept) |  |  | -0.79 | 0.36 | -2.18 | **0.029** |
| Site Suburban |  |  | 1.68 | 0.55 | 3.00 | **0.003** |
| Sex male |  |  | -0.93 | 0.39 | -2.36 | **0.018** |
| Site Suburban × Sex male |  |  | 0.84 | 0.61 | 1.38 | 0.167 |
| Comparison between Spiders and Caterpillars |  |  | Estimate | *SE* | *t* | *p*-value |
| (Intercept) |  |  | -1.52 | 0.44 | -3.48 | **< 0.001** |
| Site Suburban |  |  | -1.47 | 1.20 | -1.23 | 0.220 |
| Sex male |  |  | -1.13 | 0.47 | -2.40 | **0.016** |
| Site Suburban × Sex male |  |  | 2.80 | 1.29 | 2.17 | **0.030** |

**Table 4.** Statistics and model estimates of parental provisioning rate (A), regularity of the provisioning visits (expressed as CV of the IVIs) (B), alternation score (C) and proportion of synchronized visits (D) between rural and suburban nests. Individual estimates are given from summary statistics of the models. Models are abbreviated as follow: LM – Linear model; GLM – Generalized linear model; LMM – Linear mixed model. Significant p-values are shown in bold.

| Variable |  |  | Estimate | *SE* | *t* | *p*-value |
| --- | --- | --- | --- | --- | --- | --- |
| A*. LMM for parental provisioning rate* |  |  |  |  |  |  |
| (Intercept) |  |  | 0.84 | 0.43 | 1.97 | **0.049** |
| Site (reference rural) |  |  | -0.19 | 0.09 | -2.06 | **0.039** |
| Sex (reference female) |  |  | -0.04 | 0.04 | 0.36 | 0.352 |
| Brood size |  |  | 0.02 | 0.08 | 0.31 | 0.758 |
| *B. LMM for CV of the inter-visit intervals (IVIs)* |  |  | Estimate | *SE* | *t* | *p*-value |
| (Intercept) |  |  | 0.70 | 0.54 | 1.29 | 0.195 |
| Site (reference rural) |  |  | 0.05 | 0.11 | 0.44 | 0.656 |
| Sex (reference female) |  |  | 0.19 | 0.07 | 2.56 | **0.010** |
| Brood size |  |  | -0.03 | 0.09 | -0.31 | 0.755 |
| C. *LM for alternation score* |  |  | Estimate | *SE* | *t* | *p*-value |
| (Intercept) |  |  | 1.31 | 1.62 | 0.81 | 0.431 |
| Site (reference rural) |  |  | -0.93 | 0.34 | -2.68 | **0.016** |
| Brood size |  |  | -0.05 | 0.29 | -0.16 | 0.874 |
| D. G*LM for proportion of synchronized visits* |  |  | Estimate | *SE* | *z* | *p*-value |
| (Intercept) |  |  | -3.15 | 2.06 | -1.52 | 0.150 |
| Site (reference rural) |  |  | -1.02 | 0.44 | -2.32 | **0.036** |
| Provisioning rate |  |  | 0.04 | 0.02 | 2.29 | **0.038** |
| Proportion of visits by the male |  |  | 1.20 | 2.34 | 0.51 | 0.617 |
| Brood size |  |  | 0.34 | 0.27 | 1.24 | 0.235 |
